# Benefits and risks of napping in older adults: A systematic review

**DOI:** 10.3389/fnagi.2022.1000707

**Published:** 2022-10-21

**Authors:** Maher Souabni, Mehdi J. Souabni, Omar Hammouda, Mohamed Romdhani, Khaled Trabelsi, Achraf Ammar, Tarak Driss

**Affiliations:** ^1^Interdisciplinary Laboratory in Neurosciences, Physiology and Psychology Physical Activity, Health and Learning (LINP2), UFR STAPS (Faculty of Sport Sciences), UPL, Paris Nanterre University, Nanterre, France; ^2^Research Laboratory, Molecular Bases of Human Pathology, LR19ES13, Faculty of Medicine, University of Sfax, Sfax, Tunisia; ^3^Physical Activity, Sport and Health, UR18JS01, National Observatory of Sports, Tunis, Tunisia; ^4^Motricité-Interactions-Performance, MIP, UR4334, Le Mans Université, Le Mans, France; ^5^High Institute of Sport and Physical Education of Sfax, University of Sfax, Sfax, Tunisia; ^6^Research Laboratory: Education, Motricity, Sport and Health, EM2S, LR19JS01, University of Sfax, Sfax, Tunisia; ^7^Department of Training and Movement Science, Institute of Sport Science, Johannes Gutenberg-University Mainz, Mainz, Germany

**Keywords:** aging, sleep, napping, psychophysiological measures, cognitive and psychomotor performances, health

## Abstract

A growing body of evidence indicates that napping is common among older adults. However, a systematic review on the effect of napping on the elderly is lacking. The aim of this systematic review was to (i) determine how studies evaluated napping behavior in older adults (frequency, duration and timing); (ii) explore how napping impacts perceptual measures, cognitive and psychomotor performance, night-time sleep and physiological parameters in the elderly (PROSPERO CRD42022299805). A total of 738 records were screened by two researchers using the PICOS criteria. Fifteen studies met our inclusion criteria with a mean age ranging from 60.8 to 78.3 years and a cumulative sample size of *n* = 326. Daytime napping had an overall positive impact on subjective measures (i.e., sleepiness and fatigue), psychomotor performances (i.e., speed and accuracy) and learning abilities (i.e., declarative and motor learning). Additionally, studies showed (i) consistency between nap and control conditions regarding sleep duration, efficiency and latency, and proportion of sleep stages, and (ii) increase of 24 h sleep duration with nap compared to control condition. Based on the findings of the present review, there is minimal evidence to indicate that napping is detrimental for older adults' nighttime sleep. Future studies should consider involving repeated naps during a micro-cycle in order to investigate the chronic effect of napping on older adults.

**Systematic review registration:**

identifier: CRD42022299805.

## Introduction

Daytime napping among older adults has recently attracted the growing attention of researchers and scientists as a behavioral factor that impacts health and performance especially as diurnal napping is more prevalent and more frequent in older adults than in young and middle-aged adults (Furihata et al., 2016; Leng et al., 2016; Faraut et al., 2017; Li et al., 2017; Xiao and Hale, 2018; Yin et al., 2018; Kim et al., 2019; Yang et al., 2020). In this context, research showed that the prevalence of napping is age dependent. While only 9% of children over 5 years of age routinely take daytime naps (Komada et al., 2012). Faraut et al. (2017) reported that 40% of 14–19- year-old (yo) teenagers take daytime naps. For an older population (i.e., ≥20 yo), a Japanese study showed that only 11.7% in the young adult group (20–39 yo) reported taking daytime naps regularly (i.e., ≥4 days/week), 14.4% in the middle-age group (40–59 yo), and 25.8% in the older adult group (i.e., ≥60 yo) (Furihata et al., 2016). In Europe, a study revealed that 57.7% of the participants who reported daytime napping were older than 65 yo (Leng et al., 2016). Participants in this study were drawn from the European Prospective Investigation of Cancer-Norfolk (EPIC-Norfolk) cohort study including a total of 25,639 men and women aged 40–74 years. In addition, several studies revealed that daytime napping is flagrantly more common in middle-aged (50%) and older adults (55%) compared to other age groups among the Chinese population (Zhou et al., 2016; Li et al., 2017; Yin et al., 2018), probably due to a cultural belief, in this country, that napping promotes health (Yang et al., 2020). Similarly, a Korean-based population study reported that out of a sample of 5,427 people, 35.7 to 42.3% of its middle- to old-aged participants (40–69 years) take daytime naps (Kim et al., 2019). Furthermore, more than 300,000 middle-to-old-aged Americans were recruited in six US states (California, Florida, Louisiana, New Jersey, North Carolina, and Pennsylvania) as part of the National Institutes of Health-AARP Diet and Health Study (Xiao and Hale, 2018). The final analytic cohort included 97,890 women and 110,647 men and 40.3 to 52.6% of them reported regular daytime napping.

Importantly, sleep parameters change with aging (Ohayon et al., 2004). In adults, total sleep time and sleep efficiency decrease with age, while sleep latency and wake after sleep onset increase with age (Ohayon et al., 2004). Thus, the ability to maintain sleep decreases in older adults, which results in a shortened nocturnal sleep duration and an increased number and duration of awakenings during the night (Li et al., 2018b). In addition, the percentage of slow-wave sleep is negatively correlated with age (Ohayon et al., 2004). Therefore, sleep in older adults seems to be less consolidated due to a shortened duration of deep sleep compared to younger adults (Li et al., 2018b). These changes in sleep patterns make older adults more prone to taking naps during the daytime to compensate for the deficit of sleep during the night (Feinsilver and Hernandez, 2017). Napping in older adults is also related to other factors such as excessive daytime sleepiness, comorbidities, and medications (Zhang et al., 2020). All the evidence show that older adults with chronic health conditions (e.g., neurological disease, cardiovascular disease, cognitive impairment, insomnia, immobility, psychiatric disorders) are reported to have a higher prevalence of napping (Furihata et al., 2016; Li et al., 2018b; Liu et al., 2018; Spira et al., 2018). In this context, older adults take naps to counteract daytime sleepiness and fatigue from comorbidities. The prevalence of napping in older adults makes it of great interest to investigate the effect of napping among this population. Interestingly, studies investigating the effect of daytime napping on cognitive performance showed inconclusive results. Some studies reported an improvement of cognitive outcomes with afternoon naps (Keage et al., 2012; Li et al., 2017, 2018a). Nonetheless, other studies suggested that daytime napping may be associated with cognitive decline (Kimura et al., 2019; Leng et al., 2019).

The effect of daytime napping on performance among physically active people has been the topic of several recent studies (Botonis et al., 2021; Lastella et al., 2021; Souabni et al., 2021). Accordingly, a systematic review of the literature on the effect of daytime napping on cognitive and physical performance among older adults is warranted. The primary objectives of this paper are to (1) map out the aspects of the research, (2) outline how napping parameters can influence the potential effect on performance and health, and (3) identify gaps in the current literature.

## Methods

### Systematic review protocol

This systematic review was conducted and reported in accordance with the updated guidelines of the preferred reporting items for systematic reviews and meta-analysis statement (PRISMA), which is an evidence-based protocol describing a set of items for reporting in systematic reviews and meta-analysis (Page et al., 2021) (See Electronic Supplementary material 1 for PRISMA checklist). The study protocol was prospectively registered (PROSPERO ID: CRD42022299805).

### Information sources and search strategy

A comprehensive systematic search of studies was performed electronically in four electronic scholarly databases, namely MEDLINE, Web of Science, SPORDiscus and PubMed, from inception to December 2021. Search strategies were developed in collaboration with an information specialist (KT). Searches identified papers focused on naps or napping in older-aged populations and contained keywords relating to cognitive and physical performance (See Electronic Supplementary material 2 for Database search strategies).

To identify additional studies not included in these search terms, the reference lists of the included manuscripts were checked, as well as the related citations from other articles *via* Google Scholar and the authors' personal files. Specialists in the field were also contacted for information about possible pending publications. Additionally, target journals (i.e., Sleep, Sleep Medicine, Nature and Science in Sleep, Journal of the American Geriatric Society, Sleep and Biological Rhythms, Journal of Sports Sciences, British Journal of Sports Medicine, Chronobiology International) were hand-searched for relevant accepted studies. Definitions of key terms used in this systematic review are provided in Table 1.

**Table 1:** Terms used in this review.

**Term**	**Definition**
Time in bed (TIB)	The time elapsed between first getting into bed to the final arising.
Total sleep time (TST)	The total amount of time spent asleep whilst in bed.
Sleep efficiency (SE)	TST expressed as a percentage of TIB: TST/TIB x 100. Whether derived from instrumental measures or subjective estimates (of TST), SE provides a sensitive metric for estimating sleep quality.
Sleep inertia	A transient state between sleep to full awake during which performance is temporarily impaired. It disappears ~1 h after waking.
Post-lunch dip	A phenomenon induced by circadian rhythms characterized by a dip in performance for some variables during mid-afternoon hours.
Excessive daytime sleepiness	Excessive daytime sleepiness was defined as the inability to stay alert and awake during the day accompanied by a feeling of sleepiness.

### Eligibility criteria/selection criteria

Eligibility was set in accordance with the PICOS criteria [population: older adults (i.e., ≥55 years of age); intervention: acute and/or chronic daytime napping protocol; comparison: napping intervention vs. control and varied frequency and duration of napping interventions; outcomes: cognitive functions and/or physical performances and study design: randomized controlled trials (RCTs)].

#### Study selection

**Figure 1:**
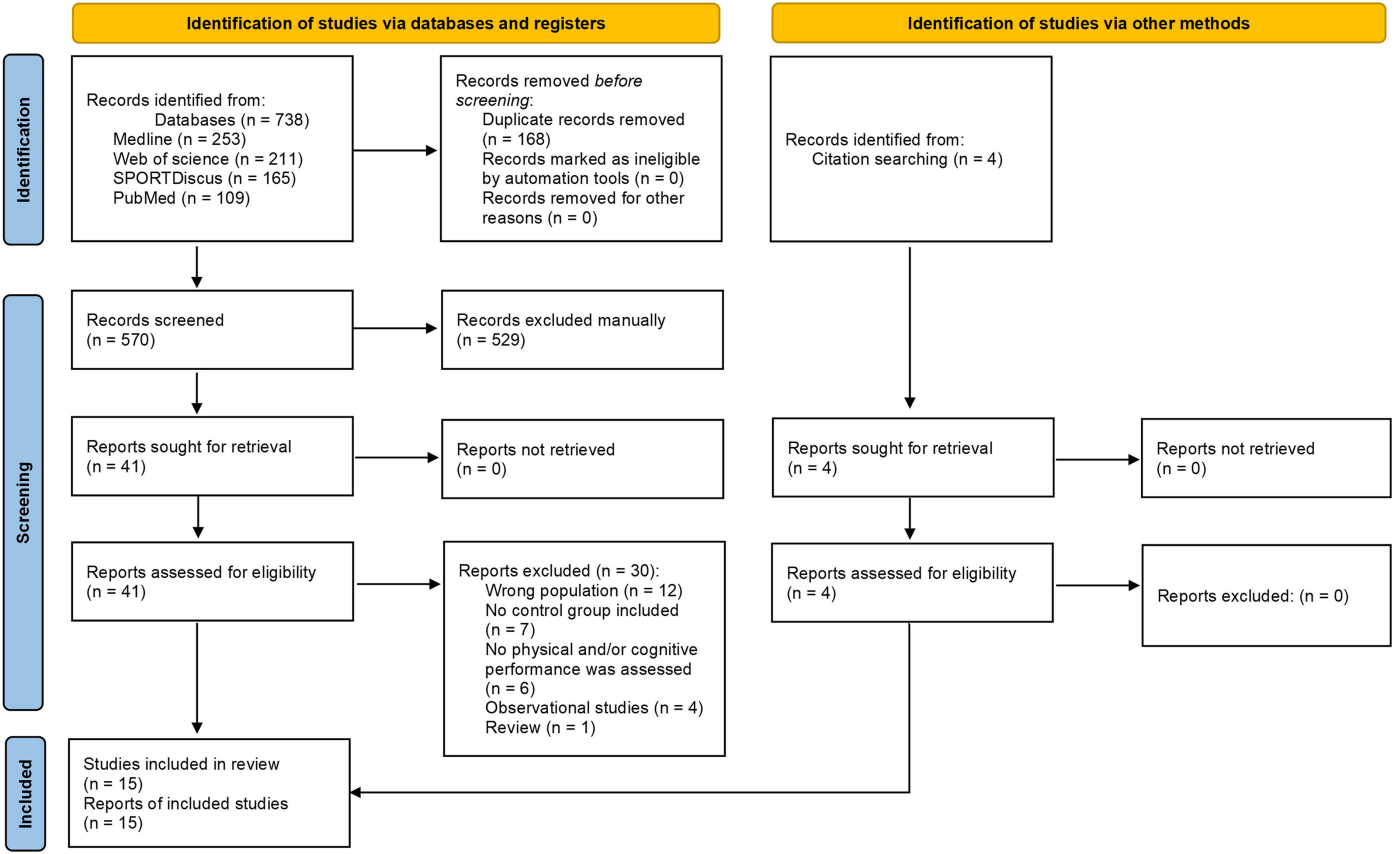
PRISMA 2020 flow diagram for new systematic reviews which included searches of databases, registers and other sources.

The process used for selecting articles is outlined in Figure 1. Following the removal of duplicates manually by MS, titles and abstracts of recovered records were reviewed independently by two authors (MS and MJS). Articles were marked “include,” “exclude” or “uncertain” according to the prespecified eligibility criteria. Selected papers (i.e., “include” and “uncertain”) were then independently read in full by two authors (MS and MJS) to finalize eligibility or exclusion. The reason for excluding an article during the full-text review was recorded (see Electronic Supplementary material 3 for excluded full-text articles). Discrepancies during title/abstract or full-text screening were resolved by a third author (TD) if there was no resolution after discussion between the two screening authors.

#### Data extraction

Using a standardized form, data were extracted independently by two reviewers (MS and MJS). Any discrepancies were identified and resolved through discussion or by involving a third reviewer (TD). The extracted data included publication details (authors surname, publication year, country), participants' characteristics (number of participants, age, sex, etc.), study design (duration, timing and frequency of daytime napping), napping measurement (actigraphy, polysomnography, self-report), and key outcomes.

### Methodological quality and risk of bias

The quantitative assessment tool “QualSyst” (Kmet et al., 2004) was used to assess the risk of bias of each study. QualSyst contains 14 items (Table 2) that are scored depending on the degree to which specific criteria are met (yes = 2, partial = 1, no = 0). Items not applicable to a particular study design were marked as “NA”. A summary score was calculated for each article by summing up the total score obtained across relevant items and dividing that by the total possible score. Quality assessments were performed by two authors (MR and MS) independently, and disagreements were solved by consensus or by the intervention of a third reviewer (OH) when necessary. Studies with a score of ≥75% were considered as of strong quality, those rated at 55–75% as of moderate quality, and a score <55% was judged as of weak quality. The percentage of lost points for each item was also calculated.

**Table 2:** Quality assessment of the studies.

	**Question described**	**Appropriate study design**	**Appropriate subject selection**	**Characteristics described**	**Random allocation**	**Researchers blinded**	**Subjects blinded**	**Outcome measures well defined and robust to bias**	**Sample size appropriate**	**Analytic methods well described**	**Estimate of variance reporter**	**Controlled for confounding**	**Results reported in detail**	**Conclusion supported by results?**	**Rating (%)**	**Study quality**
Creighton (1995)	Yes	No	Partial	Partial	No	No	No	Yes	No	No	No	Partial	Yes	Yes	39.2	Weak
Tamaki et al. (1999)	Yes	Yes	Yes	Partial	No	No	No	Yes	No	Yes	Partial	Partial	Yes	Yes	60.7	Moderate
Tamaki et al. (2000)	Yes	Yes	Yes	Partial	No	No	No	Yes	No	Yes	Partial	Partial	Yes	Yes	60.7	Moderate
Monk et al. (2001)	Yes	Yes	Yes	Yes	Yes	No	No	Yes	No	Yes	Partial	Yes	Yes	Yes	75	Strong
Campbell et al. (2005)	Yes	Yes	Yes	Yes	Yes	Yes	Yes	Yes	Yes	Yes	Partial	Partial	Yes	Yes	92.8	Strong
Milner and Cote (2008)	Yes	Yes	Yes	Yes	No	No	No	Yes	No	Yes	Yes	Yes	Yes	Yes	71.4	Moderate
Fogel et al. (2014)	Yes	Yes	Yes	Yes	Partial	No	No	Yes	Yes	Yes	Partial	Yes	Yes	Yes	78.5	Strong
Korman et al. (2015)	Yes	Yes	Yes	Yes	Partial	No	No	Yes	Yes	Partial	Partial	Yes	Partial	Yes	71.4	Moderate
Backhaus et al. (2016)	Yes	Yes	Yes	Partial	Yes	Yes	No	Yes	Yes	Yes	Partial	Yes	Yes	Yes	85.7	Strong
Baran et al. (2016)	Yes	Partial	Yes	Yes	Yes	No	No	Yes	Partial	Yes	Partial	Yes	Yes	Yes	75	Strong
King et al. (2017)	Yes	Yes	Yes	Yes	Partial	No	No	Yes	Yes	Yes	Partial	Partial	Yes	Yes	75	Strong
Heim et al. (2017)	Yes	Partial	Yes	Yes	Yes	No	No	Yes	Partial	Partial	Partial	Partial	Yes	Yes	67.8	Moderate
Scullin et al. (2017)	Yes	Yes	Yes	Yes	Partial	Yes	No	Yes	Yes	Yes	Partial	Yes	Yes	Yes	85.7	Strong
Fang et al. (2021)	Yes	Yes	Yes	Yes	Partial	No	No	Yes	Yes	Yes	Partial	Yes	Yes	Yes	78.5	Strong
Fitzroy et al. (2021)	Yes	Yes	Yes	Yes	Partial	No	No	Yes	Yes	Yes	Partial	Yes	Yes	Yes	78.5	Strong
% of lost points (%)	0	13.3	3.3	13.3	46.6	80	93.3	0	40	13.3	50	20	3.3	0	-	-

## Results

### Study selection

The predefined search strategies yielded a preliminary pool of 738 possible papers, 41 of which remained after duplicates had been excluded and titles and abstracts had been screened.

After a careful review of the 41 full texts, 30 papers were excluded (12 studies with the wrong population, seven in which no control group was included, six in which no physical and/or cognitive performance was assessed, four observational studies and one review). Eleven articles were included, as well as four additional records identified through the screening of the references and related citations from other journals *via* Google Scholar lists of included articles.

In total, 15 studies met our inclusion criteria for determining the effects of napping on cognitive and/or physical performances in older adults.

### Study characteristics

Studies characteristics are presented in Table 3. These studies were published between 1995 and 2021, arranged by order of publication date. Five studies were conducted in the USA (Creighton, 1995; Campbell et al., 2005; Baran et al., 2016; Scullin et al., 2017; Fitzroy et al., 2021), five in Canada (Monk et al., 2001; Milner and Cote, 2008; Fogel et al., 2014; King et al., 2017; Fang et al., 2021), two in Japan (Tamaki et al., 1999, 2000), two in Germany (Backhaus et al., 2016; Heim et al., 2017), and one in Israel (Korman et al., 2015). Included studies focused on the effects of napping on perceptual measures [i.e., subjective sleepiness (Creighton, 1995; Tamaki et al., 1999, 2000; Milner and Cote, 2008; Fogel et al., 2014; Backhaus et al., 2016; Fang et al., 2021; Fitzroy et al., 2021), subjective fatigue (Tamaki et al., 1999, 2000; Milner and Cote, 2008) and subjective alertness (Monk et al., 2001)], reaction time (Creighton, 1995; Tamaki et al., 1999; Monk et al., 2001; Milner and Cote, 2008; Backhaus et al., 2016), memory (Milner and Cote, 2008) and psychomotor performance (Monk et al., 2001; Campbell et al., 2005; Fogel et al., 2014), declarative (Backhaus et al., 2016; Baran et al., 2016; Heim et al., 2017) and motor (Fogel et al., 2014; Korman et al., 2015; Backhaus et al., 2016; King et al., 2017; Fang et al., 2021; Fitzroy et al., 2021) learning, nighttime sleep (Monk et al., 2001; Campbell et al., 2005; Korman et al., 2015; King et al., 2017) and physiological parameters (Tamaki et al., 1999; Monk et al., 2001).

**Table 3:** Studies characteristics (population, study design and napping parameters).

**Author, year, country**	**Participants**	**Experimental design**	**Nap(s) duration, timing, frequency, assessment method**
Creighton (1995) USA	6 (>75 y) (1 M, 5 F) m = NM	Single-subject methodology was used to study the responses of each subject individually. An ABA (reversal) design was applied.	90 min 13:00 h 5 days NM
Tamaki et al. (1999) Japan	6 (66–78 y) m = NM	Subjects participated in both conditions with an interval of 1 week.	30 min 13:00 h 1 day EEG
Tamaki et al. (2000) Japan	10 (66–78 y) m = NM	Ten healthy elderly persons who habitually napped in the afternoon three or more times a week participated in the present study.	30 min 13:00 h 1 day EEG
Monk et al. (2001) Canada	9 (74–87 y) (4 M, 5 F) m = 78.3 y	Order of the two conditions was counterbalanced: 4 subjects experienced nap condition followed by the no-nap condition, 5 the reverse order.	90 min 13:30 h 17 days−14 D Actigraphy (Home)−3 D PSG (Lab)
Campbell et al. (2005) USA	32 (55–85 y) (16 M, 16 F) m = 68.5 y SD = 8.1 y	Two-session, within-subject laboratory design. Order determined by the flip of a coin before the first laboratory visit.	120 min 14:00 h 1 day EEG
Milner and Cote (2008) Canada	12 (56–70 y) (7 M, 5 F) m = 61 y SD = 5 y	Conditions were counterbalanced across participants.	60 / 20 min Timing of naps varied across individuals according to their individual sleep times. 1 day EEG
Fogel et al. (2014) Canada	30 (55–75 y) (10 M, 20 F) m = 62.6 y SD = 5.0 y	15 NAP 15 CON	90 min 13:00 h 1 day PSG
Korman et al. (2015) Israel	21 (60–75 y) (11 M, 10 F) m = 64.8 y SD = 4.3 y	11 NAP 10 CON	90 min NM 1 day PSG
Backhaus et al. (2016) Germany	33 (60–82 y) Sequence learning (*n* = 33) -wake 11 F/M (9/2) m(SD) 73.7(4.5) -short nap: 12 F/M (7/5) m(SD) 69.9(6.1) -long nap: 10 F/M (5/5) m(SD) 71.3(6.0) Motor adaptation (*n* = 30) -wake: 10 F/M (9/1) m(SD) 74.2(4.5) -short nap: 10 F/M (5/5) m(SD) 69.3(6.7) -long nap: 10 F/M (6/4) m(SD) 71.1(5.5)		45/90 min NM 1 day PSG
Baran et al. (2016) USA	13 (60–75 y) (3 M, 10 F) m = 67 y SD = 3.4 y	Conditions were counterbalanced across participants.	120 min NM 1 day PSG
King et al. (2017) Canada	31 (≥55 y) -nap: 15 F/M (13/2) m(SD) 69.9(1.2) -rest: 16 F/M (13/3) m(SD) 63.5(1.4)		90 min 13:00 h 1 day PSG
Heim et al. (2017) Germany	30 (50–75 y) -nap: 10 F/M (6/4) m(SD) 62.6(1.8) -rest: 10 F/M (7/3) m(SD) 59.9(1.6) -interfering activity: 10 F/M (5/5) m(SD) 60.0(1.5)		90 min NM 1 day NM
Scullin et al. (2017) USA	45 (58–83 y) -nap: 29 F/M (15/14) m(SD) 69.69(7.1) -rest: 16 F/M (10/6) m(SD) 70.13(7.8) Participants were randomly assigned to either the nap or quiet wakefulness conditions, in a 3:2 ratio. The intent of this approach was to increase statistical power for PSG correlational analyses if a significant effect of nap/wake condition was observed.		90 min 14:00 h 1 day PSG
Fang et al. (2021) Canada	30 (55–75 y) (9 M, 21 F) m = 62.6 y SD = 5.0 y	15 NAP 15 CON	90 min 13:00 h 1 day PSG
Fitzroy et al. (2021) USA	18 (58–75 y) (10 M, 8 F) m = 65.39 y SD = 5.80 y	Conditions were counterbalanced across participants.	120 min 13:00 h 1 day HD-PSG *High-density polysomnography*

M, male; F, female; m, mean; NM, not mentioned.

### Methodological quality and risk of bias

Quality scores for the included studies ranged from 39.2% (weak) to 92.8% (strong). Most studies (*n* = 9) were rated as strong quality, five were of moderate quality and one of weak quality. Causes of lost points included subjects blinded (93.3%), researchers blinded (80%) and random allocation (46.6%) (Table 2).

### Subjects' characteristics

The studies involved in this systematic review included a total of 326 participants. Except for two studies (Tamaki et al., 1999, 2000) in which gender was not mentioned (*n* = 16), studies included 120 males and 190 females. The number of participants in each trial ranged from 6 (Creighton, 1995; Tamaki et al., 1999) to 45 (Scullin et al., 2017), with a mean sample size of 21.73 (SD 12.08). Mean age ranging from 60.8 (Heim et al., 2017) to 78.3 (Monk et al., 2001) years.

### Effect of napping on perceptual measures

#### Subjective sleepiness

Eight (53.33%) studies (Creighton, 1995; Tamaki et al., 1999, 2000; Milner and Cote, 2008; Fogel et al., 2014; Backhaus et al., 2016; Fang et al., 2021; Fitzroy et al., 2021) have focused on the effects of napping on subjective sleepiness with inconclusive results (Table 4). Four studies showed that sleepiness was not affected by napping opportunities [i.e., 45 (Backhaus et al., 2016), 90 (Fogel et al., 2014; Backhaus et al., 2016; Fang et al., 2021) and 120 min (Fitzroy et al., 2021)]. Otherwise, it has been reported that a 30-min nap opportunity (NAPO) decreased sleepiness compared to the control condition (Tamaki et al., 1999, 2000). Interestingly, Milner and Cote (2008) reported that a 60-min NAPO decreased sleepiness but not a 20-min NAPO. Moreover, Creighton (1995) showed contradictory outcomes between subjects. Out of the six participants, four felt drowsier, with sleepiness scores rising during nap week compared to control week. Nonetheless, a fifth participant displayed the opposite response (Creighton, 1995).

**Table 4:** The effects of napping on perceptual measures.

**Measured parameter**	**Author, year**	**Nap(s) duration**	**Nap(s) timing**	**Nap(s) frequency**	**Effects of napping**
Subjective sleepiness	Creighton (1995)	90 min	13:00 h	5 days	4 ↑ 1 ↓
	Tamaki et al. (1999)	30 min	13:00 h	1 day	↓
	Tamaki et al. (2000)	30 min	13:00 h	1 day	↓
	Milner and Cote (2008)	20 min	See Table 2	1 day	↔
		60 min			↓
	Fogel et al. (2014)	90 min	13:00 h	1 day	↔
	Backhaus et al. (2016)	Short nap 45 min Long nap 90 min	NM	1 day	↔
	Fang et al. (2021)	90 min	13:00 h	1 day	↔
	Fitzroy et al. (2021)	120 min	13:00 h	1 day	↔
Subjective fatigue	Tamaki et al. (1999)	30 min	13:00 h	1 day	↓
	Tamaki et al. (2000)	30 min	13:00 h	1 day	↓
	Milner and Cote (2008)	20 min	See Table 2	1 day	↓
		60 min			↓
Subjective alertness	Monk et al. (2001)	90 min	13:30 h	7 days (home)	↔
				2 days (lab)	↔

NM, not mentioned.

#### Subjective fatigue

Three (20%) studies (Tamaki et al., 1999, 2000; Milner and Cote, 2008) investigated the effect of napping on subjective fatigue. Results showed that daytime naps had a positive impact on subjective fatigue. Milner and Cote (2008) reported that participants rated themselves as less fatigued following both a 60- and a 20-min NAPO. Similarly, two studies (Tamaki et al., 1999, 2000) revealed that a 30-min NAPO reduced significantly subjective fatigue.

#### Subjective alertness and wellbeing

The effect of a 90-min NAPO on alertness and wellbeing was investigated during 17 days (14 days at home and 3 days in the laboratory) (Monk et al., 2001). Nine visual analog scales—yielding scores of global vigor (alertness) and global affect (wellbeing)—were presented to participants four times per day. Results showed a consistency between nap and control conditions in self-rated evening alertness at home (66 vs. 65, *P* > 0.25) which was also evident (71 vs. 70, *P* > 0.25) in the laboratory data.

### Effect of napping on cognitive and psychomotor performance

Studies focused on the effect of napping on reaction time (Creighton, 1995; Tamaki et al., 1999; Monk et al., 2001; Milner and Cote, 2008; Backhaus et al., 2016), memory (Milner and Cote, 2008) and psychomotor (Monk et al., 2001; Campbell et al., 2005; Fogel et al., 2014) performance with inconclusive results (Table 5). The effect of a 30-min NAPO on reaction time was investigated using a visual detection task (Tamaki et al., 1999). Results showed that reaction time was shorter in nap conditions than in rest conditions, and that the percentage of correct responses increased after taking a nap, but decreased after taking a rest (Tamaki et al., 1999). Importantly, Campbell et al. (2005) showed that a 120-min NAPO was associated with several significant improvements in cognitive and psychomotor performance using a Walter Reed Performance Assessment Battery. This consists of four tasks: the two-letter visual search task, the Wilkinson four-choice reaction time task, the logical reasoning task and the Stroop congruency task. Output measurements from the performance tasks included accuracy, speed and a summary measure [i.e., throughput = (accuracy x speed)/100]. Overall better performance was observed for the same day (average of 5 p.m. and 7 p.m. trials) results after NAPO. A significant improvement of throughput for Wilkinson four-choice reaction time and Stroop task (*P* < 0.05, Δ = 15.5%; *P* < 0.05, Δ = 9.4%, respectively) and speed during Stroop task (*P* < 0.03, Δ = 9.2%) was reported in the nap condition compared to control condition. Further, the improvement in throughput on the reaction time task was positively correlated with amounts of Stage 4 sleep (*P* < 0.03, *r* = 0.41) and Stages 3 and 4 combined (*P* < 0.05, *r* = 0.36) obtained during the nap. Regarding the Stroop task, enhanced throughput and speed were positively associated with sleep period time of naps (*P* < 0.05; *r* = 0.39 and 0.40, respectively). Furthermore, next day performance (average of 6 trials from 9 a.m. to 7 p.m.) was measured on the same study (Campbell et al., 2005) and results showed the same improvement for the Stroop task's speed (*P* < 0.02, Δ = 6.5%) and throughput (*P* < 0.02, Δ = 6.6%) compared to control condition. In addition, a significantly better performance was reported for the logical reasoning task (i.e., accuracy, *P* < 0.03, Δ = 2.5%) and the two-letter search task (i.e., throughput, *P* < 0.03, Δ = 4.7%). Moreover, improvements in speed and throughput on the Stroop were significantly correlated with longer sleep times (*r* = 0.44 and 0.42, respectively; *P* < 0.02). The increased accuracy on the logical reasoning task was positively correlated with nap duration (*r* = 0.42; *P* < 0.05), sleep efficiency (*r* = 0.40; *P* < 0.05), and Stage 2 amounts (*r* = 0.56; *P* < 0.01). It is noteworthy that sex did not affect the outcome of performance measures.

**Table 5:** The effects of napping on cognitive and psychomotor performances.

**Author, year**	**Measured performances**	**Nap(s) duration**	**Nap(s) timing**	**Nap(s) frequency**	**Effects of napping**
Creighton (1995)	Eye-hand reaction time	90 min	13:00 h	5 days	↔
	Symbol Digit Modalities Test (Subjects are asked to decode a line of symbols according to a key at the top of the worksheet)				2 ↑ 4 ↔
Tamaki et al. (1999)	Reaction time. visual detection task;	30 min	13:00 h	1 day	↑
	Percentage of correct responses. Visual detection task				↑
Monk et al. (2001)	Multiple Sleep Latency Test. As a measure of objective evening sleeping	90 min	13:30 h	2 days (lab)	↑ mean sleep latency (from 11.5 to 15.6 min) → reduced objective evening sleepiness in the nap condition.
	Visual vigilance hits				↔
	Pegboard latency				↔
	Four-choice serial response				↔
	Commission errors made in the response inhibition task				↔
Campbell et al. (2005)	Walter Reed Performance Assessment Battery. -The two-letter visual search task, -The Wilkinson four-choice reaction time task, -The logical reasoning task, -The Stroop congruency task. Output measures from the performance tasks included accuracy, speed and throughput [(accuracy x speed)/100].	120 min	14:00 h	1 day	Session 1 same day (average of 5 p.m. and 7 p.m.) ↑ 15.5% Throughput The Wilkinson four-choice reaction time task. ↑ 9.2% Speed ↑ 9.4% Throughput The Stroop congruency task.
					Session 2 next day (average of 6 trials from 9 a.m. to 7 p.m.) ↑ 4.7% Throughput The two-letter visual search task, ↑ 2.5% Accuracy The logical reasoning task, ↑ 6.5% Speed ↑ 6.6% Throughput The Stroop congruency task.
Milner and Cote (2008)	Accuracy on simple reaction test	20 min	See Table 2	1 day	↔
	Reaction time on simple reaction test				↔
	Serial addition/subtraction task	60 min			↔
	Working memory				↔
Fogel et al. (2014)	Psychomotor Vigilance Task	90 min	13:00 h	1 day	↔
Backhaus et al. (2016)	Simple reaction time	45 min 90 min	NM	1 day	↔

NM, not mentioned; ↑, better performance compared to control condition; ↔, no significant difference between nap and control condition.

Interestingly, a beneficial effect of naps (90-min NAPO) was observed on objective alertness during a multiple sleep latency test (Monk et al., 2001). Mean sleep latency increased significantly from 11.5 to 15.6 min (*P* < 0.01) indicating a reduction in objective evening sleepiness in the nap condition. However, another study (Fogel et al., 2014) did not report any significant effect on objective sleepiness measured using a psychomotor vigilance task for the same NAPO (90 min). Moreover, a symbol digit modalities test (i.e., a neuropsychological test that measures the ability to concentrate on a cognitive task) was performed following the same NAPO (90 min) and results were inconclusive (Creighton, 1995). Two of the six subjects demonstrated better performance during the nap phase of the study compared to the control phase. In the same study (Creighton, 1995), the nap did not appear to affect performance during the eye-hand reaction time test in which participants were asked to respond as quickly as possible to a flashing red light by pushing a button. Further studies investigated the effect of napping on reaction time using various NAPO [i.e., 20 (Milner and Cote, 2008), 45 (Backhaus et al., 2016), 60 (Milner and Cote, 2008), 90 min (Monk et al., 2001; Backhaus et al., 2016)] but results did not reveal any significant effect of napping on performance.

Only one study (Milner and Cote, 2008) investigated the effect of daytime napping on memory. Milner and Cote (2008) used a 2-back memory test to investigate the effect of a 20- and 60- min NAPO on working memory. Participants were asked to identify target letters (i.e., when the displayed letter matches one seen two letters previously: “a,” “R,” “a”) during three blocks of 60 randomly presented letters on a screen. No significant difference was reported in performance between the nap and control conditions. Therefore, from this perspective, memory performances seem to not be affected by napping of both durations (i.e., 20 and 60 min) in older adults.

### Effect of napping on learning

Nine (60%) studies (Fogel et al., 2014; Korman et al., 2015; Backhaus et al., 2016; Baran et al., 2016; Heim et al., 2017; King et al., 2017; Scullin et al., 2017; Fang et al., 2021; Fitzroy et al., 2021) investigated the effect of daytime napping on declarative (Backhaus et al., 2016; Baran et al., 2016; Heim et al., 2017) and motor (Fogel et al., 2014; Korman et al., 2015; Backhaus et al., 2016; King et al., 2017; Fang et al., 2021; Fitzroy et al., 2021) learning capacities in older adults (Table 6). Overall, the selected studies used a range of designs and methodological approaches. It should be noted that Backhaus et al. (2016) examined both declarative and motor learning.

**Table 6:** The effects of napping on learning.

**Type of learning**	**Author, year**	**Measured performances**	**Nap(s) duration**	**Nap(s) timing**	**Nap(s) frequency**	**Effects**
**Declarative learning**	Baran et al. (2016)	Word pair learning task. Stimuli consisted of single-syllable, concrete nouns that were paired to create two lists of 40 semantically unrelated cue-target word pairs (e.g., bath–grass).	120 min	See Table 2	1 days	↔
	Backhaus et al. (2016)	An auditory presentation of 15 words. Retained knowledge of the list of words was tested.	45 min 90 min	NM	1 day	↔
	Heim et al. (2017)	Pseudo-word learning task. The task consisted of memorizing monsters' names (a 1- to 3-syllabic pseudo-word in German) while presented visually (for 10 sec each) on the screen and aurally (twice) *via* loudspeakers.	90 min	NM	1 day	↑ 18.1% in re-test 1 vs. test
						↑ 20.3% in re-test 2 vs. test
	Scullin et al. (2017)	Declarative learning was assessed using two measures: free recall test (participants were given 5 min to write down all the words they studied) and Recognition test (participants viewed the 100 “old” studied words and 100 “new” lure words).	90 min	14:00 h	1 day	↔
**Motor learning**	Fogel et al. (2014)	Finger-tapping task. Participants were instructed to perform a five-item sequence (4-1-3-2-4).	90 min	13:00 h	1 day	↔
	Korman et al. (2015)	Finger-tapping task. Participants were trained to generate a given five-element finger-to-thumb opposition sequence (4-1-3-2-4) with their non-dominant left hand.	90 min	13:00 h	1 day	↔
						↑ same day (8-h later)
						↑ next day (22-h later)
	Backhaus et al. (2016)	Finger-tapping task. Participants were trained to generate a nine-element sequence using the four fingers of the left hand on a lap top keyboard with covers.	45 min 90 min	NM	1 day	↔
	King et al. (2017)	Finger tapping task. during a test-retest protocol initial learning phase at the pre-nap session, at 11:00 h, and retests were administered 8 and 22 h later.	90 min	13:00 h	1 day	↔
						↑ same day (8-h later)
						↑ next day (22-h later)
	Fitzroy et al. (2021)	Serial reaction time task. participants were informed that cues would be sequential during the indicated blocks and instructed to notice and learn any patterns they could.	120 min	13:00 h	1 day	↔
						↑ same day (5-h later)

NM, not mentioned.

#### Declarative learning

Four studies (Backhaus et al., 2016; Baran et al., 2016; Heim et al., 2017; Scullin et al., 2017) examined the effect of napping on declarative learning with inconclusive results. Only one of the four studies demonstrated a positive impact of napping on language learning using a pseudo-word learning task (Heim et al., 2017). The task consisted of memorizing monsters' names (a 1- to 3-syllabic pseudo-word in German) that are presented visually (for 10 s each) on the screen and aurally (twice) *via* loudspeakers. Performance was re-tested twice: the first re-test took place on the same day after rest or a nap while the second re-test took place 24 h afterwards. Statistical results revealed a significant increase in language learning scores from test to re-test 1 (*P* = 0.01, Δ = +18.1%) and re-test 2 (*P* = 0.01, Δ = +20.3%) following a 90-min NAPO. However, in rest conditions, language learning scores decreased significantly between the test and re-test 1 (*P* = 0.003, Δ = −66.3%). Baran et al. (2016) used the word pair learning task on a computer (programmed using E-Prime) following a 120-min NAPO to investigate the effect of napping on declarative learning. Stimuli consisted of single-syllable, concrete nouns that were paired to create two lists of 40 semantically unrelated cue-target word pairs (e.g., bath–grass, rail–bag) (Baran et al., 2016). In another study, declarative learning was assessed using two measures (i.e., free recall and recognition tests) (Scullin et al., 2017). For the free recall test, participants were given 5 min to write down all the words they studied. Next, they completed a recognition test on the computer in which they viewed the 100 “old” studied words and 100 “new” lure words. Participants were instructed to indicate which words were old words (i.e., studied words) and which were new words (i.e., non-studied words). On the other hand, a different approach was reported for Backhaus et al. (2016) using only an auditory presentation of 15 words. There, retained knowledge of the list of words was tested twice (i.e., same and next day) (Backhaus et al., 2016). In the last three studies (Backhaus et al., 2016; Baran et al., 2016; Scullin et al., 2017), participants were not found to learn differently as a function of the prescribed sleep condition (i.e., nap or rest).

#### Motor learning

Studies investigating the effect of NAPO on motor learning performance showed inconclusive results (Fogel et al., 2014; Korman et al., 2015; Backhaus et al., 2016; King et al., 2017; Fang et al., 2021; Fitzroy et al., 2021). All the studies used a finger tapping task to assess motor/procedural learning performance with some methodological differences. Additionally, it is important to mention that Fogel et al. (2014) and Fang et al. (2021) are both part of the same large study and presented the same results concerning learning performances.

The positive impact of napping was evident in three of the five studies examining the effects of napping on motor learning performances. In Korman et al. (2015)'s study, participants were trained to generate a given five-element finger-to-thumb opposition sequence (4-1-3-2-4) with their non-dominant left hand. The same protocol was used previously to investigate the effect of daytime napping on motor sequence learning (MSL) in younger adults (Korman et al., 2007). Performance was tested four times; a pre-nap test (learning session) and 3 re-tests (0, 8, and 22 h post-training). The affordance of a 90-min NAPO immediately after training resulted in robust overall gains in speed (*F*_3,30_ = 21.1, *P* < 0.001), within-session (*F*_1,10_ = 22.9, *P* = 0.001) and overnight (*F*_1,10_ = 7.97, *P* = 0.018). There was also no deterioration of performance at 8 h post-training (*F*_1,10_ = 0.8, *P* = 0.389). Nonetheless, for the control condition, an overall improvement in speed performance (*F*_3,27_ = 34.35, *P* < 0.001) was reported, but there was no significant gain in speed overnight and the 8 h post-training performance speed tended to decline from that attained by the end of the training session (0 h, 8 h post-training, *F*_1,9_ = 5.39, *P* = 0.072). Similarly, King et al. (2017) used a finger tapping task during a test-retest protocol (initial learning phase at the pre-nap session, at 11:00 h, and re-tests were administered 8 and 22 h later) to assess the effect of a 90-min NAPO on MSL performance. Importantly, although performance did not differ between control and nap groups during the initial learning phase, a beneficial effect of napping was reported across same and next day re-tests. This beneficial effect was consistent across the two follow-up sessions, and thus the positive impact of the daytime nap lasted for at least 22 h in older adults. In the same way, motor learning was compared prior to the nap and wake intervals in Fitzroy et al. (2021) study using a different task. During a serial reaction time task, participants were informed that cues would be sequential during the indicated blocks and instructed to notice and learn any patterns they could. Results showed that across-interval performance improvement in older adults was significant for the nap condition but not for the wake condition.

On the other hand, Fogel et al. (2014) and Fang et al. (2021) reported that MSL was not affected by napping (i.e., 90-min NAPO). Results of the study suggest that both nap and control groups had a similar performance during the initial learning phase of the motor skill (at 11:00 h) and re-test session (at 16:00 h). Indeed, performance improved from the training to the re-test session irrespective of the sleep/wake condition. Similarly, Backhaus et al. (2016) reported that both, 45- and 90-min NAPO, did not affect MSL performance.

### Effect of napping on nocturnal sleep

Four (26.66%) studies (Monk et al., 2001; Campbell et al., 2005; Korman et al., 2015; King et al., 2017) have focused on the effect of napping on subsequent nighttime sleep (Table 7). The general pattern of results indicates that napping did not negatively impact nocturnal sleep. Three studies (Campbell et al., 2005; Korman et al., 2015; King et al., 2017) compared sleep on the night immediately after a nap with sleep immediately after the control condition. Campbell et al. (2005) revealed that there was no significant difference between both conditions (120-min NAPO/rest) in total sleep time (TST), sleep efficiency (SE) and the minutes or proportion of any sleep stage. However, sleep onset latency (SOL) in the nap condition compared to the control condition was longer: on average, participants took 6.3 min longer to fall asleep. Moreover, when the 24-h period containing the nap was compared with the 24-h control condition, participants averaged more than an hour more in TST (7.4 h vs. 6.2 h). Accordingly, the amount and proportion of stage 1, stage 2 and rapid eye movement sleep (REM) increased significantly when a nap was taken. In the same way, Korman et al. (2015) and King et al. (2017) reported that a 90-min NAPO did not affect the subsequent nighttime sleep; the two experimental groups did not differ in TST, SE, SOL. Additionally, King et al. (2017) reported that there was no difference between conditions in amount and proportion of stage 1, stage 2, REM and slow-wave sleep (SWS). The 24-h TST in the nap condition was not compared with the rest condition for both studies.

**Table 7:** The effects of napping on nighttime sleep.

**Author, year**	**Nap(s) duration**	**Nap(s) timing**	**Nap(s) frequency**	**Effects of napping**
Monk et al. (2001)	90 min	13:30 h	7 days (home)	↔ TST ↔ TIB ↔ SE ↔ SOL ↔ Sleep quality ↔ bedtime ↔ waketime ↔ WASO ↑ 24-h TST
			2 days (lab)	↓ TST ↓ TIB ↓ SE ↔ SOL ↔ bedtime ↓ waketime ↔ WASO ↔ REM
**Campbell et al. (** **2005** **)**	120 min	14:00 h	1 day	↔ TST ↔ SE ↑ SOL ↑ 24-h TST ↔ Minutes/% Stage 1 ↔ Minutes/% Stage 2 ↔ Minutes/% Stage 3 and 4 ↔ Minutes/% REM
Korman et al. (2015)	90 min	13:00 h	1 day	↔ TST ↔ SE ↔ SOL
King et al. (2017)	90 min	13:00 h	1 day	↔ TST ↔ SE ↔ SOL ↔ minutes/% Stage 1 ↔ minutes/% Stage 2 ↔ minutes/% SWS ↔ minutes/% REM

NM, not mentioned; REM, rapid eye movement; SE, sleep efficiency; SOL, sleep onset latency; SWS, slow wave sleep; TIB, time in bed; TST, total sleep time; WASO, wake after sleep onset.

Further, Monk et al. (2001) examined the effect of a 90-min NAPO on night-time sleep during 17 days (14 days at home and 3 days in the laboratory). Wrist actigraphy (average of the second week of home study) showed that there was no significant difference between conditions in TST, TIB, SE, sleep quality, bedtime, waketime and wake after sleep onset (WASO). Interestingly, 24 h TST was higher in nap compared to the control condition (7.3 vs. 6.6 h, respectively). However, the average of nights 2 and 3 in the laboratory (measured *via* polysomnography) revealed a significant decrease in the nap condition compared to the control condition for TST (5.3 vs. 6.1 h, respectively), TIB (7.1 vs. 7.9 h, respectively), SE and waketime. Moreover, no significant difference was reported for WASO, SOL, REM sleep and bedtime.

### Effect of napping on physiological parameters

Only two (13.3%) studies examined the effect of daytime napping on physiological parameters. Blood pressure was recorded before and after nap and control condition (Tamaki et al., 1999). Results showed that diastolic blood pressure decreased following NAPO compared to rest. Further, core body temperature was measured continuously in Monk et al. (2001)'s study to analyze the effect of a 90-min NAPO on circadian variations across 24 h. A slight dip in the temperature was reported between 13:30 and 15:00 h due to the nap; however, there was no difference in circadian phases of temperature between the nap and control groups.

## Discussion

This systematic review is the first to explore (1) how daytime naps could impact perceptual measures (e.g., sleepiness, fatigue and alertness), cognitive and psychomotor performance, declarative and motor learning, and nighttime sleep in older adults and, (2) how napping parameters (frequency, duration, timing and measurement) could potentially influence the effect of napping. This information is important for older adults and geriatric specialists who provide advice and education to the elderly regarding napping for health improvement.

### Why, when and how much older adults nap?

Studies reported a high prevalence of daytime napping in older adults as a result of several factors: health conditions, medications, excessive daytime sleepiness, boredom, lack of physical activity and changes in sleep patterns implying lower night-time sleep quantity and quality. Further, it is well known that diurnal sleepiness induced by circadian rhythms occurs in the afternoon (Mitler et al., 1988; Broughton and Mullington, 1992; Monk, 2005; Waterhouse et al., 2007). This phenomenon is called post-lunch dip and is characterized by a dip in performance for some variables during mid-afternoon hours (Monk, 2005), especially for people who have been partially deprived of sleep (Romdhani et al., 2019). In this context, several studies proposed mid-afternoon as the best time to onset nap (Waterhouse et al., 2007; Romdhani et al., 2021; Souabni et al., 2021). In the same way, all the studies included in the present systematic review initiated the NAPO in the mid-afternoon between 13:00 and 14:00 h. Regarding the nap duration, most studies employed 90 min as a duration for NAPO. This duration was present in 9 (60%) out of the 15 studies included in this systematic review. Three studies (Campbell et al., 2005; Baran et al., 2016; Fitzroy et al., 2021) reported a duration of 120 min for NAPOs and the remaining studies (*n* = 3) reported NAPOs with lower durations [i.e., 20 (Milner and Cote, 2008), 30 (Tamaki et al., 1999, 2000) and 60 min (Milner and Cote, 2008)]. In addition, in a recent systematic review by our team, we suggested 90 min as an optimal NAPO for athletes undergoing chronic sleep deprivation (Souabni et al., 2021). The reason was twofold: for one, it is postulated that rapid eye movement (REM) sleep has a vital role in restorative benefits for cognition (Belenky et al., 2003; Hobson, 2005) and is also associated with memory consolidation and learning of motor skills (Davenne, 2009; Venter, 2012), while non-rapid eye movement (NREM) sleep is when the body actively repairs and restores itself (Davenne, 2009; Venter, 2012). Further, slow wave sleep (SWS)—also known as deep NREM sleep—is thought to play an important role in cerebral restoration and recovery (Dijk, 2009; Wisor et al., 2013). Moreover, a 90-min nap duration allows—in theory—a complete sleep cycle (NREM + REM) to occur (Davies et al., 2010) and consequently could reduce the severity of sleep inertia, since REM sleep is a lighter sleep state and waking up from this sleep stage is easier (Ferrara and De Gennaro, 2000). This could be a possible explanation for the choice of this duration for older adults.

### Was performance improved by daytime napping?

Regarding perceptual measures, a positive effect of napping was reported for sleepiness and fatigue. Only Creighton (1995) reported a negative impact of napping on sleepiness. Out of six participants, four felt an increase in sleepiness following a nap and one felt the opposite effect. Further, Monk et al. (2001) revealed that alertness and vigor were not affected by naps. It is important to mention that both studies investigated the chronic effect of naps with participants adopting a napping regimen involving a 90-min NAPO each day (Creighton, 1995; Monk et al., 2001). Thus, the results were taken on an average of 5 (Creighton, 1995) and 7 (Monk et al., 2001) days. As for the study of Creighton (1995), inconsistency in providing the scheduled naps was presented as a possible explanation to the contradictory results.

Interestingly, a positive impact of napping was reported on cognitive and psychomotor performance on the same (Tamaki et al., 1999; Campbell et al., 2005) and the following day (Campbell et al., 2005). There is no evidence to indicate that napping is detrimental to older adults' cognitive and psychomotor performance. Again, there was great variability in the effect of repeated naps during a micro-cycle. Two of the six subjects demonstrated better performance in attention during the nap phase of the study compared to the control phase (Creighton, 1995). On the other hand, naps did not appear to affect performance during eye-hand reaction time (Creighton, 1995). Similarly, Milner and Cote (2008) reported a beneficial effect of the nap on objective alertness during a multiple sleep latency test, while reaction time was not affected during multiple tasks. The chronic effect of napping is yet unclear, but it was reported that habitual nappers displayed lighter sleep inertia upon awakening compared to non-habitual nappers (Dinges, 1992). By the same reasoning, a possible explanation is that the effect of naps might change with repeated naps every day, and—depending on participants—might gradually decrease. Further studies are needed to bring insight regarding the effect of habitual napping in older adults. Otherwise, work memory performance was tested following two NAPO durations (i.e., 20- and 60-min) in the same study (Milner and Cote, 2008), and the change in performance between napping and not napping did not significantly differ. A plausible explanation could be that both durations were not sufficient to impact memory performance. Future work should investigate the effect of longer nap durations (e.g., 90 min) on working memory performance.

Based on the findings of the present review, daytime napping had an overall positive impact on learning performances. A nap benefitted vocabulary learning in older individuals and this effect persisted to the next day (Heim et al., 2017). Similarly, procedural/motor learning was positively impacted by daytime napping. Although performance did not differ between control and nap groups during the initial learning phase (pre-nap), a beneficial effect of naps was reported across the same (Korman et al., 2015; King et al., 2017; Fitzroy et al., 2021) and next-day (Korman et al., 2015; King et al., 2017) re-tests following a 90-min NAPO. This beneficial effect was consistent across the follow-up sessions and lasted for at least 22 h in older adults (Korman et al., 2015; King et al., 2017). However, this beneficial effect of napping was not observed in the large study of Fogel et al. (2014) and Fang et al. (2021) with the same NAPO duration. Discrepancies between studies could be related to the differences in methodological approaches and protocols used. Indeed, MSL was assessed using the same task for the studies (Fogel et al., 2014; Korman et al., 2015; King et al., 2017; Fang et al., 2021), while learning performance was tested only once (i.e., same day) for Fogel et al. (2014) and Fang et al. (2021), and it was retested twice (i.e., same and next day) for Korman et al. (2015) and King et al. (2017). In addition, same-day re-tests took place at different moments [i.e., 5 (Fogel et al., 2014; Fang et al., 2021) and 8 (Korman et al., 2015; King et al., 2017) h later from the initial learning session]. We believe that if tests took place later in the same day or even the next day, a possible significant effect of napping could be observed in MSL. Also, a different motor learning task was adopted in the Fitzroy et al. (2021) study, where participants performed an explicit variant of the serial reaction time task. Here, they were made aware that there was an underlying pattern in the stimulus sequence, but they were not directly informed what that pattern was. Specifically, participants were only informed that cues would be sequential during the indicated blocks and instructed to notice and learn any patterns they could. This could explain the beneficial effect of napping even when retested 5 h later from the initial learning session. Furthermore, Backhaus et al. (2016) did not reveal any significant effect of daytime napping on MSL performance neither the same nor the next day. It is important to mention that this study aimed to investigate whether sleep-dependent consolidation can be elicited in diurnal settings in MSL using a more difficult task (i.e., 9-item sequence task). Therefore, it could be argued that the beneficial effect of daytime napping—regarding MSL performance—seems to be less effective with difficult tasks.

### Was nocturnal sleep affected by daytime napping?

Studies investigating acute (Campbell et al., 2005; Korman et al., 2015; King et al., 2017) and chronic (Monk et al., 2001) effects of daytime napping on nighttime sleep showed consistency between nap and control conditions regarding TST (Monk et al., 2001; Campbell et al., 2005; Korman et al., 2015; Slater et al., 2015), SE (Monk et al., 2001; Campbell et al., 2005; Korman et al., 2015; Slater et al., 2015), SOL (Korman et al., 2015; King et al., 2017) and proportion of sleep stages (Campbell et al., 2005; King et al., 2017). Additionally, 24 h TST was higher in nap compared to control condition (Monk et al., 2001; Campbell et al., 2005; King et al., 2017). Based on the results of the present review, there is minimal evidence to indicate that napping is detrimental for older adults' nighttime sleep. Importantly, it was reported that SOL was 6.3 min longer in the nap condition compared to control condition following a 120-min NAPO (Campbell et al., 2005). Further, Monk et al. (2001) showed a decrease in TST, SE and wake time (in that the participant woke up earlier) following naps. It is important to point out that those results were taken from an average of two nights in the laboratory, which is an unfamiliar place to older adults, compared to the first part of the study which took place in their beds at home. The study also used polysomnography, which can be a complex and uncomfortable technique for the participants (Vlahoyiannis et al., 2020). All these factors might affect older adults' sleep and therefore could explain the decrease of TST in nap condition.

### Methodological considerations

Out of 15, only two (13.3%) studies (Creighton, 1995; Monk et al., 2001) reported a napping regimen lasting more than 1 day. Participants adopted a napping regimen (i.e., older adults took a nap each day) involving a 90-min NAPO lasting 5 (Creighton, 1995) and 17 (Monk et al., 2001) days. Clearly, a tendency toward a great variability in results was observed. Therefore, future investigations may involve repeated naps during a micro-cycle to explore the chronic effect of napping on older adults. Further, all studies investigating motor learning implemented solely hand movements (finger sequence tapping tasks). It would be of interest to investigate the effect of daytime napping on movements implementing the whole body. One of the positive points to mention is that electroencephalography-based devices were used in 13 (86.6%) of the 15 studies included in the present systematic review which—in spite of the fact that it is a complex and uncomfortable technique—gives a clear insight into sleep stages and more precise sleep duration and efficiency (Scott et al., 2020; Vlahoyiannis et al., 2020).

It is well established that adequate sleep is vital for optimal cognitive and physical functioning across the lifespan (Carskadon, 2011; Pace-Schott and Spencer, 2011; Lo et al., 2012). Nonetheless, almost half of older adults reported at least one sleep problem (Neikrug and Ancoli-Israel, 2010). A systematic review and meta-analysis of studies relating to sleep duration and cognition in older adults showed that short (≤5 h) and long (≥9 h) sleep are associated with cognitive impairment (Lo et al., 2016). These associations were found in both cross-sectional and prospective studies, and across multiple cognitive domains (e.g., executive functions, verbal memory, working memory capacity, etc.). Furthermore, a recent study conducted by Wang et al. (2019) included a total of 161,241 participants aged 35–70 years from 21 countries with different income levels in seven geographic regions (North America and Europe, South America, the Middle East, South Asia, Southeast Asia, China, and Africa), confirmed the above-mentioned conclusions. In this study, the authors concluded that (i) estimated total sleep duration of 6–8 h per day is associated with the lowest risk of deaths and major cardiovascular events and (ii) daytime napping is associated with increased risks of major cardiovascular events and deaths in those with >6 h of nighttime sleep but not in those sleeping ≤6 h/night (Wang et al., 2019). In the present systematic review, a 90-min duration was adopted as a nap opportunity in 9 (60%) out of the 15 studies. This NAPO has been proved to be the optimal nap duration—in view of the above-mentioned reasons—for athletes carrying sleep debt (Souabni et al., 2021). Similarly for older adults, this nap duration is deemed successful to improve cognitive and psychomotor performance, and declarative and motor learning. However, a longer NAPO (i.e., 120 min) seems to have an impact on subsequent nighttime sleep (i.e., longer sleep latency). Moreover, longer daytime napping was associated with a higher risk for diabetes mellitus, odds ratios were 1.23 for those reporting <60 min and 1.55 for those reporting >60 min of napping compared with individuals who did not nap (Xu et al., 2011). Similarly, another study including a total of 27,009 retired workers reported that longer nap duration (i.e., >60 min) may represent a novel risk factor for diabetes mellitus and higher blood glucose levels (Fang et al., 2013). In addition, a recent study aiming to examine the association between daytime napping and successful aging on 7,469 participants reported that the group having long daytime naps (i.e., >60 min/day) was associated with a lower probability of achieving successful aging compared with the one having no daytime naps (i.e., 0 min/day) (Xin et al., 2020). All those factors should be taken into consideration to draw a firm conclusion regarding the adequate duration of nap for the elderly. A comprehensive recording of sleep duration and quality of older adults seems to be necessary. Specifically, these studies must use objective methods of sleep assessment (e.g., polysomnography, actigraphy and heart rate variability) and consider a duration of at least 7 days for a reliable measure to assess habitual sleep patterns in older adults.

## Strengths and limitations

This is the first systematic review of the literature on the effects of napping on older adults' perceptual, cognitive and physical measures. The strengths of the present analysis include a comprehensive coverage of the available literature and a careful appraisal of its quality. The databases PubMed, Web of Science, Scopus, SPORTDiscus and Google Scholar were searched for studies regardless of the time when they were conducted and the languages they were published in. However, this current systematic review has limitations which should be acknowledged. First, the number of articles that include napping regimens (*n* = 2) was low, and there were reservations with compliance with the protocol in one of the studies (Creighton, 1995). Second, both studies did not have sufficient data on nap quality. For example, Creighton (Creighton, 1995) reported neither quantity nor quality of sleep during the nap, and Monk et al. (2001) declared only TST during the nap. It is absolutely necessary for future studies to provide quantitative (e.g., TST and duration and amount of each sleep stage) and qualitative (e.g., sleep efficiency and/or fragmentation indices) data examining napping behaviors of older adults in order to ensure that medical doctors and researchers are able to comprehensively examine the chronic effects of naps in the elderly. Finally, meta-analyses were not able to be conducted owing to the low number of studies of each type of measured variables.

## Conclusion

**Figure 2:**
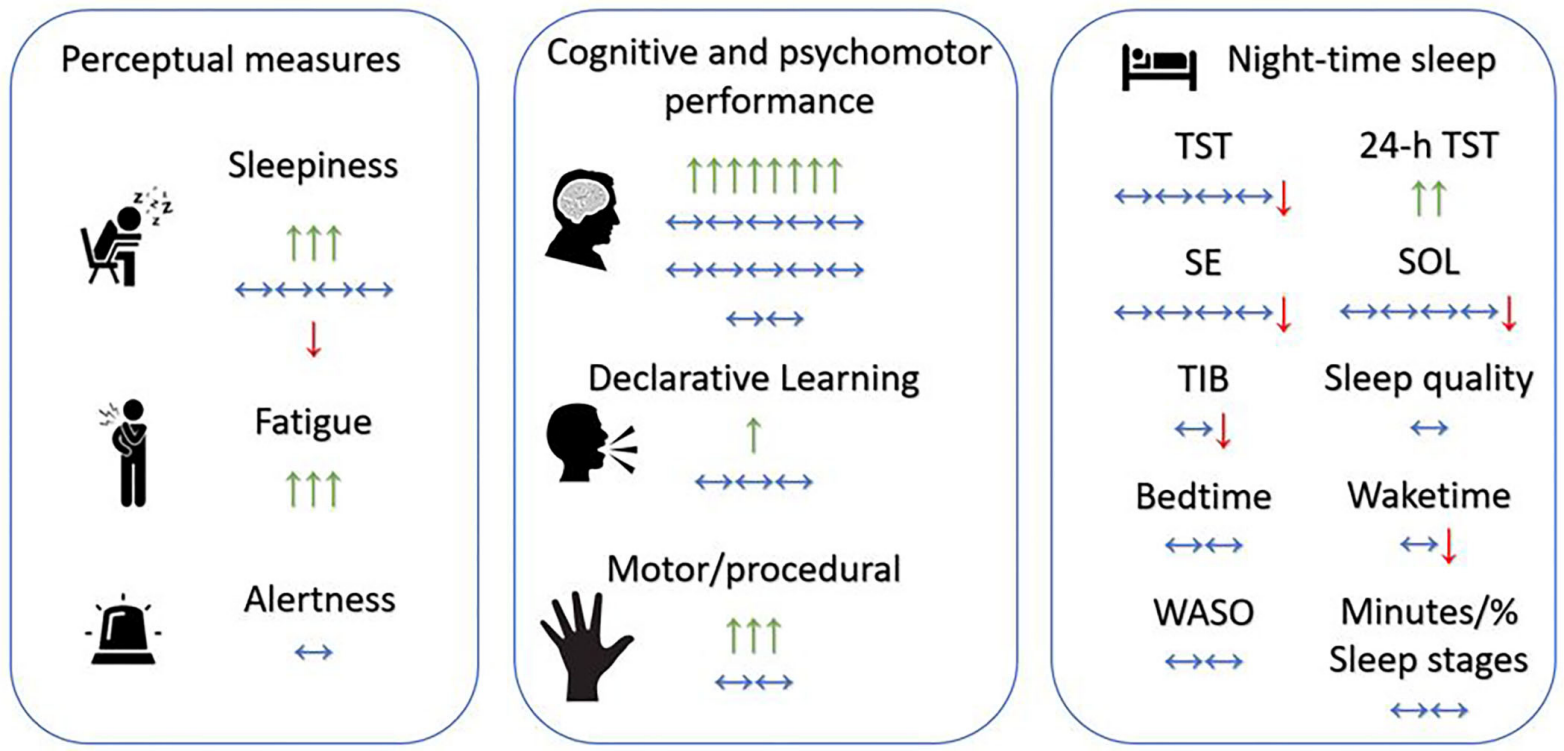
Overview of the effects of daytime napping on older adults. Green arrow up ↑, positive significant effect of one study/one nap duration (e.g., positive effect on sleepiness/fatigue means nap reduced sleepiness/fatigue); Red arrow down ↓, negative significant effect of one study/one nap duration (e.g., negative effect on sleepiness means nap increased sleepiness); horizontal-two-edged arrow ↔, no significant effect of one study/one nap duration; SE, sleep efficiency; SOL, sleep onset latency; TIB, time in bed; TST, total sleep time; WASO, wake after sleep onset.

The findings of the present systematic review are meaningful for understanding the impact of daytime napping on the life of older adults. Because short sleep is associated with cognitive impairment in older adults across ages, and given the huge beneficial effect of napping on perceptual measures, cognitive and psychomotor performance, and declarative and procedural learning (Figure 2), a diurnal daytime nap opportunity could be proposed as a solution to improve older adults' health and daily performance. Nonetheless, considering the long-term impact of long sleep on cognitive performance, researchers, geriatric specialists and doctors should be careful about which nap duration to propose to older adults in order to not exceed the recommended duration of sleep. Finally, while napping is a common practice among senior populations, older adults should be aware of the importance of good sleep hygiene so that they could establish good sleep habits and steer clear of extreme sleep durations.

## Data availability statement

The original contributions presented in the study are included in the article/Supplementary material, further inquiries can be directed to the corresponding author.

## Author contributions

MS drafted the manuscript. MS, MJS, and TD selected retrieved relevant papers. MR, MS, and OH assessed studies' qualities. OH, MR, KT, AA, and TD critically reviewed the manuscript. AA and TD were the guarantors of the overall content. All authors have read and agreed the submitted version.

## Conflict of interest

The authors declare that the research was conducted in the absence of any commercial or financial relationships that could be construed as a potential conflict of interest.

## Publisher's note

All claims expressed in this article are solely those of the authors and do not necessarily represent those of their affiliated organizations, or those of the publisher, the editors and the reviewers. Any product that may be evaluated in this article, or claim that may be made by its manufacturer, is not guaranteed or endorsed by the publisher.
