# Supplementary material for: Benefits and risks of napping in older adults: A systematic review
Source: Front Aging Neurosci. 2022 Oct 21;14:1000707. doi: 10.3389/fnagi.2022.1000707 (PMC9634571; doi:10.3389/fnagi.2022.1000707)
Supplement: Supplementary file 1 [file Data_Sheet_1.zip › Revised Supplementary file 1, 2 and 3/Revised_Electronic Supplementary Material 2.docx]

Electronic Supplementary Material 2: Database search strategies

Medline

*Searched:* 16/12/2021

TS=(nap OR napping OR "daytime nap*" OR "diurnal nap*")

AND TS=(aged OR "older adult*" OR "elderly adult*" OR "older individual*" OR "elderly individual*")

AND TS=("cognitive performance" OR "cognitive outcome" OR "decision making" OR "mental process" OR "reaction time" OR "psychomotor performance" Or vigilance OR alertness OR "physical performance" OR "physical outcomes" OR "athletic performance" OR strength OR agility OR speed OR endurance OR "aerobic performance" OR "anaerobic performance")

PubMed

*Searched:* 15/12/2021

("Nap"[Text Word] OR "napping"[Text Word] OR "diurnal napping"[Text Word] OR "daytime nap*"[Text Word])

AND ("Aged"[MeSH Terms] OR "older adult*"[Text Word] OR "elderly individual*"[Text Word] OR "elderly*"[Text Word])

AND ("Cognition"[MeSH Terms] OR "Decision Making"[MeSH Terms] OR "Reaction Time"[MeSH Terms] OR "Mental Processes"[MeSH Terms] OR "cognitive performance*"[Text Word] OR "psychomotor performance*"[Text Word] OR "alertness*"[Text Word] OR "Reaction Time"[Text Word] OR "vigilance"[Text Word] OR "Athletic Performance"[MeSH Terms] OR "Sports"[MeSH Terms] OR "endurance*"[Text Word] OR "speed*"[Text Word] OR "agility"[Text Word] OR "aerobic performance*"[Text Word] OR "anerobic performance*"[Text Word] OR "strength*"[Text Word])

SPORTDiscus

*Searched:* 14/12/2021

“TX ( naps or napping or "diurnal nap*" or "daytime nap*" )

AND TX ( "older adult*" or "elderly adult*" or "older individual*" )

AND TX ( "cognitive performance" or "cognitive outcome*" or "reaction time" or "executive function*" or vigilance or alertness or memory or "psychomotor performance*" or "physical performance*" or "athletic performance*" or endurance or speed or "aerobic performance*" or anaerobic performance*" )”

Web of Science

*Searched:* 14/12/2021

“ALL=(nap OR napping OR "daytime nap*" OR "diurnal nap*")

AND ALL=(aged OR "older adult*" OR "elderly adult*" OR "older individual*" OR "elderly individuals*")

AND ALL=("cognitive performance" OR "cognitive outcomes" OR "decision making" OR "mental process" OR "reaction time" OR "psychomotor performance" Or vigilance OR alertness OR "physical performance" OR "physical outcomes" OR "athletic performance" OR stregth OR agility OR speed OR endurance OR "aerobic performance" OR "anaerobic performance")”
