# Supplementary material for: Benefits and risks of napping in older adults: A systematic review
Source: Front Aging Neurosci. 2022 Oct 21;14:1000707. doi: 10.3389/fnagi.2022.1000707 (PMC9634571; doi:10.3389/fnagi.2022.1000707)
Supplement: Supplementary file 1 [file Data_Sheet_1.zip › Revised Supplementary file 1, 2 and 3/Revised_Electronic Supplementary Material 3.docx]

Electronic Supplementary Material 3: Full-text articles excluded

| Wrong population (n = 12) | No control group included (n = 7) | No physical and/or cognitive performance was assessed (n = 6) | Observational studies (n = 4) | Review (n = 1) |
| --- | --- | --- | --- | --- |
| Harma et al. (1989)  “Daytime napping and its effects on alertness and short-term memory performance in shift workers.” | Campbell et al. (2011)  “Effects of a month-long napping regimen in older individuals” | Aber & Webb (1986)  “Effects of a limited nap on night sleep in older subjects.” | Basta et al. (2020)  “Objective Daytime Napping is Associated with Disease Severity and Inflammation in Patients with Mild to Moderate Dementia1” | Takahashi (2003)  “The role of prescribed napping in sleep medicine” |
| DOI: [10.1007/BF00409390](https://doi.org/10.1007/bf00409390) | DOI: [10.1111/j.1532-5415.2010.03264.x](https://doi.org/10.1111/j.1532-5415.2010.03264.x) | DOI: [10.1037//0882-7974.1.4.300](https://doi.org/10.1037/0882-7974.1.4.300) | DOI: [10.3233/JAD-190483](https://doi.org/10.3233/jad-190483) | DOI:  [10.1053/smrv.2002.0241](https://doi.org/10.1053/smrv.2002.0241) |
| Rogers & Aldrich (1993)  “The effect of regularly scheduled naps on sleep attacks and excessive daytime sleepiness associated with narcolepsy” | Westerberg et al. (2015)  “Memory improvement via slow-oscillatory stimulation during sleep in older adults.” | Hayward et al. (1992)  “Neuropsychological functioning and sleep patterns in the elderly.” | Theadom et al. (2015)  “Daytime napping associated with increased symptom severity in fibromyalgia syndrome” |  |
| PMID: 8455986 | DOI: [10.1016/j.neurobiolaging.2015.05.014](https://doi.org/10.1016/j.neurobiolaging.2015.05.014) | DOI: [10.5694/j.1326-5377.1992.tb121609.x](https://doi.org/10.5694/j.1326-5377.1992.tb121609.x) | DOI: [10.1186/s12891-015-0464-y](https://doi.org/10.1186/s12891-015-0464-y) |  |
| Gillberg et al. (1996)  “The effects of a short daytime nap after restricted night sleep” | Cordi et al. (2015)  “Improving sleep and cognition by hypnotic suggestion in the elderly.” | George et al. (2009)  “Sleep quality and falls in older people living in self- and assisted-care villages” | Li et al. (2018)  “Intermediate, But Not Extended, Afternoon Naps May Preserve Cognition in Chinese Older Adults.” |  |
| DOI: [10.1093/sleep/19.7.570](https://doi.org/10.1093/sleep/19.7.570) | DOI: [10.1016/j.neuropsychologia.2015.02.001](https://doi.org/10.1016/j.neuropsychologia.2015.02.001) | DOI: [10.1159/000146786](https://doi.org/10.1159/000146786) | DOI: [10.1093/gerona/glx069](https://doi.org/10.1093/gerona/glx069) |  |
| Vgontzas et al. (2007)  “Daytime napping after a night of sleep loss decreases sleepiness, improves performance, and causes beneficial changes in cortisol and interleukin-6 secretion” | Ladenbauer et al. (2016)  “Brain stimulation during an afternoon nap boosts slow oscillatory activity and memory consolidation in older adults.” | Nguyen-Michel et al. (2015)  “Underperception of Naps in Older Adults Referred for a Sleep Assessment: An Insomnia Trait and a Cognitive Problem.” | Hu et al. (2021)  “Sleep, inflammation and cognitive function in middle-aged and older adults A population-based study.” |  |
| DOI: [10.1152/ajpendo.00651.2005](https://doi.org/10.1152/ajpendo.00651.2005) | DOI: [10.1016/j.neuroimage.2016.06.057](https://doi.org/10.1016/j.neuroimage.2016.06.057) | DOI: [10.1111/jgs.13660](https://doi.org/10.1111/jgs.13660) | DOI: [10.1016/j.jad.2021.02.013](https://doi.org/10.1016/j.jad.2021.02.013) |  |
| Schweitzer et al. (2006)  “Laboratory and field studies of naps and caffeine as practical countermeasures for sleep-wake problems associated with night work” | Johnson et al. (2020)  “Sensorimotor performance is improved by targeted memory reactivation during a daytime nap in healthy older adults” | Liu et al. (2018)  “The relationship between depression, daytime napping, daytime dysfunction, and snoring in 0.5 million Chinese.” |  |  |
| DOI: [10.1093/sleep/29.1.39](https://doi.org/10.1093/sleep/29.1.39) | DOI: [10.1016/j.neulet.2020.134973](https://doi.org/10.1016/j.neulet.2020.134973) | DOI: [10.1186/s12889-018-5629-9](https://doi.org/10.1186/s12889-018-5629-9) |  |  |
| Sagaspe et al. (2007)  “Aging and nocturnal driving: better with coffee or a nap? A randomized study” | Ladenbauer et al. (2021)  “Memory-relevant nap sleep physiology in healthy and pathological aging” | Bueno et al. (2019)  “Napping, functional capacity and satisfaction with life in older adults: A population-based study.” |  |  |
| DOI: [10.1093/sleep/30.12.1808](https://doi.org/10.1093/sleep/30.12.1808) | DOI: [10.1093/sleep/zsab002](https://doi.org/10.1093/sleep/zsab002) | DOI: [10.1111/jocn.14768](https://doi.org/10.1111/jocn.14768) |  |  |
| Horne et al. (2008)  “Sleep extension versus nap or coffee, within the context of 'sleep debt'” | Cross et al. (2015)  “Napping in older people 'at risk' of dementia: relationships with depression, cognition, medical burden and sleep quality.” |  |  |  |
| DOI: [10.1111/j.1365-2869.2008.00680.x](https://doi.org/10.1111/j.1365-2869.2008.00680.x) | DOI: [10.1111/jsr.12313](https://doi.org/10.1111/jsr.12313) |  |  |  |
| Dostálová et al. (2011)  “The influence of a short daytime nap and the influence of its timing on psychomotor efficiency” |  |  |  |  |
| ISSN: 12100552 |  |  |  |  |
| van Schie et al. (2014)  “The influences of task repetition, napping, time of day, and instruction on the Sustained Attention to Response Task” |  |  |  |  |
| DOI: [0.1080/13803395.2014.968099](https://doi.org/10.1080/13803395.2014.968099) |  |  |  |  |
| Gotts et al. (2015)  “The association between daytime napping and cognitive functioning in chronic fatigue syndrome” |  |  |  |  |
| DOI: [10.1371/journal.pone.0117136](https://doi.org/10.1371/journal.pone.0117136) |  |  |  |  |
| Shaikh & Coulthard (2019)  “Nap-mediated benefit to implicit information processing across age using an affective priming paradigm” |  |  |  |  |
| DOI: [10.1111/jsr.12728](https://doi.org/10.1111/jsr.12728) |  |  |  |  |
| Chen et al. (2020)  “Autonomic Activity during a Daytime Nap Facilitates Working Memory Improvement” |  |  |  |  |
| DOI: [10.1162/jocn_a_01588](https://doi.org/10.1162/jocn_a_01588) |  |  |  |  |
